# The Differential Impact of Acute Exercise and Mindfulness Meditation on Executive Functioning and Psycho-Emotional Well-Being in Children and Youth With ADHD

**DOI:** 10.3389/fpsyg.2021.660845

**Published:** 2021-06-14

**Authors:** Hannah Bigelow, Marcus D. Gottlieb, Michelle Ogrodnik, Jeffrey D. Graham, Barbara Fenesi

**Affiliations:** ^1^Faculty of Education, Western University, London, ON, Canada; ^2^Department of Kinesiology, Faculty of Science, McMaster University, Hamilton, ON, Canada; ^3^Faculty of Health Sciences, Ontario Tech University, Oshawa, ON, Canada

**Keywords:** attention-deficit hyperactivity disorder, acute exercise, psycho-emotional well-being, mindfulness meditation, executive functioning, inhibition, working memory, task switching

## Abstract

This study investigated how acute exercise and mindfulness meditation impacts executive functioning and psycho-emotional well-being in 16 children and youth with ADHD aged 10–14 (male = 11; White = 80%). Participants completed three interventions: 10 min of exercise, 10 min of mindfulness meditation, and 10 min of reading (control). Before and after each intervention, executive functioning (inhibitory control, working memory, task-switching) and psycho-emotional well-being (mood, self-efficacy) were assessed. Mindfulness meditation increased performance on all executive functioning tasks whereas the other interventions did not (*d* = 0.55–0.86). Exercise enhanced positive mood and self-efficacy whereas the other interventions did not (*d* = 0.22–0.35). This work provides preliminary evidence for how acute exercise and mindfulness meditation can support differential aspects of executive and psycho-emotional functioning among children and youth with ADHD.

## Introduction

Attention Deficit Hyperactivity Disorder (ADHD) is one of the most common neurodevelopmental disorders affecting ~6% of children worldwide (Polanczyk et al., 2014). ADHD is characterized by cognitive deficits affecting the domain of executive functioning, including impaired inhibitory control, working memory, and task-switching (Antshel et al., 2014). Deficits in executive functioning can impair mental processes that support self-regulation and can delay learning and development (Polanczyk et al., 2014). Eighty percent of children with ADHD also have a co-occurring psychological disorder, particularly in the realm of affect and mood disorders, such as anxiety and depression (Gordon-Lipkin et al., 2018). Several efficacious methods exist to treat ADHD-related impairments, including pharmacological and behavioral management treatments, yet they all have shortcomings. The use of pharmacological treatments, for instance, is associated with undesirable side-effects, including (but not limited to) loss of appetite, insomnia, irritability, abdominal pain, and fatigue (Buitelaar, 2017). Adherence to ADHD medication is low in pediatric populations largely because of these side-effects (Wang et al., 2016). Behavioral management treatments, conversely, do not induce the same adverse side effects. However, changes in symptoms brought on by these treatments may fail to generalize to other settings or behaviors and they tend to be costly in terms of time and resources (Rajwan et al., 2012). Thus, there is an ongoing search to find effective ADHD interventions that can supplement or even replace existing ones.

Both physical exercise and mindfulness meditation have emerged in recent years as potential behavioral strategies that could ameliorate ADHD symptoms and supplement alternative treatments. Indeed, engaging in long-term, regular exercise may modify and regulate the structure and functions of the brain that underlie cognition and behavior, as well as the underlying physiology present in ADHD (Pontifex et al., 2013; Cerrillo-Urbina et al., 2015). Regular exercise has been shown to improve executive functioning and academic achievement among those with and without ADHD (Cerrillo-Urbina et al., 2015; Neudecker et al., 2019; Welsch et al., 2021). Beyond the neurocognitive benefits, regular exercise improves mood, affect, emotional regulation, self-efficacy, and decreases depressive and anxiety-like symptoms in diverse populations (Peluso and Andrade, 2005). A few studies have examined the link between exercise and psycho-emotional functioning among children and adults with ADHD (Jensen and Kenny, 2004; Fritz and O'Connor, 2016) and have shown that those who participate in regular exercise show less oppositional, restless and impulsive behavior, as well as greater emotional stability. It is clear that regular exercise is typically an important aspect of a healthy lifestyle for any population; however, adherence to programs, implementation feasibility, and access to resources present challenges to this intervention method for ADHD. As such, there has been a recent focus directed toward investigating the potential benefits of acute exercise for mitigating ADHD symptomology. Acute exercise has been shown to enhance executive functioning for both neurotypical children (Chang et al., 2012) and for those with ADHD (Pontifex et al., 2013; Benzing et al., 2018; Neudecker et al., 2019), especially in domains of inhibitory control, working memory, decision making, and cognitive flexibility. In the domain of psycho-emotional well-being however, only one study has been conducted demonstrating the benefit of acute exercise on general mood states in those with ADHD, and it was conducted exclusively in young men (Fritz and O'Connor, 2016).

Mindfulness meditation likely promotes cognitive and psychological well-being in typically developing individuals and those with ADHD (Howarth et al., 2019). Rooted in Buddhist practices, mindfulness meditation emphasizes placing attentional awareness on the present moment while deliberately remaining open to feelings and emotions that arise (Kabat-Zinn, 2009). Some research suggests mindfulness meditation ameliorates executive functioning deficits associated with ADHD, such as inattention and hyperactivity (Murrell et al., 2015; Tercelli and Ferreira, 2020). This has been demonstrated in undergraduates with ADHD (Zeidan et al., 2010) and in adults and adolescents with ADHD (Zylowska et al., 2008; Van de Weijer-Bergsma et al., 2012). A recent analysis examining how mindfulness-based interventions affected children with ADHD found encouraging results (Chimiklis et al., 2018). These interventions were associated with enhanced executive functioning and on-task behavior, decreased parental stress, and improved parent-child relationships (Chimiklis et al., 2018). Mindfulness meditation has also been shown to reduce reports of depression and anxiety (Zylowska et al., 2008; Van de Weijer-Bergsma et al., 2012), and improve parent-child relationships (Tercelli and Ferreira, 2020) in ADHD populations. However, all aforementioned studies compared experienced meditators to non-meditators, and focused on mindfulness meditation interventions that were either short term (4 days to 1 week) or long term (months to years; Evans et al., 2018). There is novel evidence, however, that the length of mindfulness-based interventions does not impact the intervention's effectiveness; shorter interventions may be just as efficacious as longer alternatives for reducing ADHD symptomology (Vekety et al., 2021). Engagement in just one session of a mindful activity (i.e., mindful coloring) is associated with increased mindfulness (Carsley and Heath, 2018). Despite this initial evidence, there has not been enough rigorous research conducted (e.g., studies using active control groups and an adequate sample size) to make definitive commentary about how acute mindfulness meditation affects children and youth with ADHD (Chimiklis et al., 2018; Tercelli and Ferreira, 2020; Vekety et al., 2021).

Although prior evidence supports the efficacy of week-long or month-long physical exercise or mindfulness meditation programs in mitigating ADHD symptoms (Cornelius et al., 2017; Chimiklis et al., 2018; Vekety et al., 2021), the effect of acute bouts (i.e., single, short bouts) in supporting children and youth with ADHD remains unclear (Colzato et al., 2016; Vekety et al., 2021). Establishing the effectiveness of an acute bout of exercise and mindfulness meditation could advance their applications as potential interventions, providing caregivers and individuals with ADHD additional easily accessible tools to reduce ADHD symptoms in just 10-min. Thus, the current study aimed to establish how an acute bout of exercise and mindfulness meditation could support executive functioning and psycho-emotional well-being in children and youth with ADHD. Specifically, we aimed to address key gaps in the literature by furthering our understanding of how acute exercise and mindfulness meditation impacts executive functioning and psycho-emotional well-being in children and youth with ADHD.

Given the above, the present study sought to directly compare the efficacy of these two behavioral interventions (i.e., exercise and mindfulness meditation) in children and youth with ADHD. While there are similarities between them (e.g., they are low-cost behavioral alternatives to pharmacological approaches), and they both show promise in ameliorating the struggles children and youth with ADHD experience, it is important to determine the differential effects of each intervention to maximize their practical applications (e.g., determining whether one intervention is superior for increasing mood-states). Although some prior research has compared exercise and mindfulness mediation, only a few studies have evaluated the relative contributions of exercise and mindfulness meditation in supporting executive functioning and alleviating ADHD symptomology. However, these studies have either been systematic reviews with no direct empirical comparison of the two interventions (Herbert and Esparham, 2017), or have been conducted exclusively in neurotypical younger adults (Van Der Zwan et al., 2015; de Bruin et al., 2016; Luu and Hall, 2017; Edwards et al., 2018) or neurotypical older adults (Håkansson et al., 2017). Prior research has also primarily involved chronic engagement in interventions and programs, especially in the area of mindfulness meditation, but there has been minimal work investigating the impact of acute bouts. To our knowledge, the present study is one of the first to assess the efficacy of just one meditation session on symptoms of ADHD in children. Most importantly, no research to date has evaluated the respective efficacy of exercise and mindfulness meditation among children and youth with ADHD. The current study used a pre- post-test, within-subjects design to assess the effects of a 10-min bout of moderate intensity exercise vs. a 10-min bout of mindfulness meditation (vs. 10 min of a reading control) on the executive functioning and psycho-emotional well-being of children and youth with ADHD aged 10–14.

Based on the research described above, it was predicted that acute exercise and acute mindfulness meditation would improve core executive functions of inhibitory control, working memory, and task-switching, along with psycho-emotional well-being in the domain of mood and general self-efficacy in children and youth with ADHD when compared to silent reading. Given that there has been no prior work empirically evaluating the relative efficacy of these two interventions within this population, there were no directional predictions made as to which intervention would yield stronger outcomes.

## Materials and Methods

### Participants

The study sample included 16 children with ADHD. A sample size calculation was performed using G*Power (version 3.1.9.6 Faul et al., 2009) with a large effect size Cohens *d* = 1.09 (Pontifex et al., 2013; Drollette et al., 2014; Luu and Hall, 2017), power of 0.80, alpha of 0.05, with primary outcome variables as changes in executive functioning and mood, indicating 16 participants were needed. All participants were between 10 and 14 years old (*M* = 11.38; *SD* = 1.5), had an ADHD diagnosis (confirmed both verbally and via questionnaire), and were recruited from London, Ontario (Canada) community clinics through paper and e-mail advertisements between June 2019 and January 2020. There were 11 males and 5 females in our sample, reflecting the common discrepancy in ADHD diagnosis with males diagnosed more often than females (Polanczyk et al., 2014). Participants were excluded if they were not fully literate or did not speak English, if they had any neurological or developmental exceptionalities beyond ADHD, and if they were color-blind (as it would interfere with their performance on executive functioning tasks). Sample demographics are provided in Supplementary Table 1. The study was approved by Western University's Research Ethics Board. Guardians provided informed written consent and children provided informed written assent before participating in the study.

### Design

**Figure 1:**
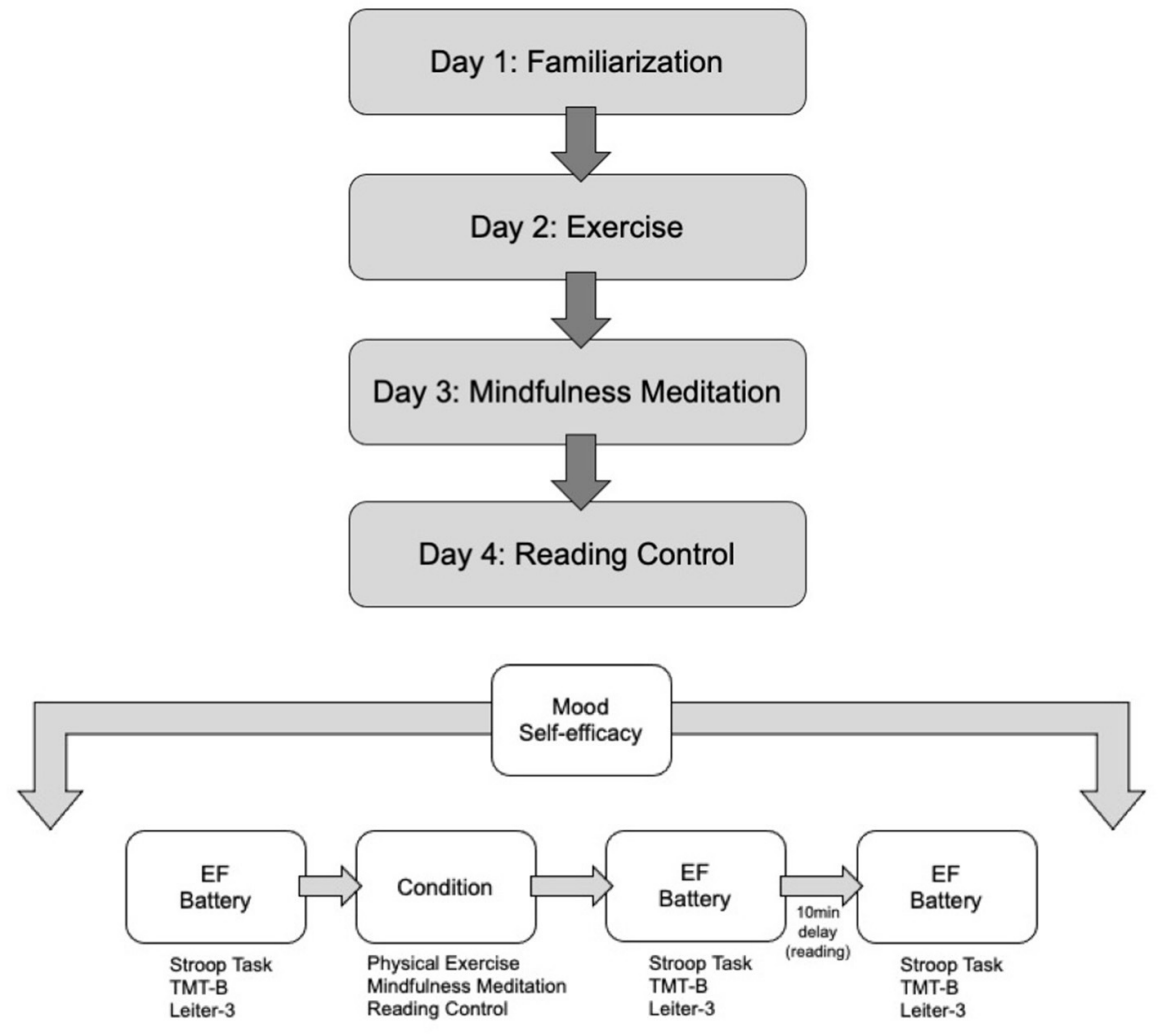
Flow diagram of study. **(Top)** Flow of study. Participants engaged in all 4 days of activities and the days were counterbalanced among participants (e.g., some participants engaged in reading control on Day 2; some engaged in mindfulness meditation on Day 1, etc.). **(Bottom)** Flow of study days. On each day of the study (other than familiarization), participants completed mood and self-efficacy measures, followed by an executive functioning (EF) battery, followed by a study condition, followed by an immediate post-test EF battery, followed by 10 min of silent reading and ended the study session with a delayed post-test EF battery.

The study utilized a pre- post-test, counterbalanced within-subjects design wherein each participant participated in all conditions in a random order. This design is considered the “gold-standard” for testing acute effects of exercise on executive functioning (Pontifex et al., 2019). The experiment consisted of four sessions separated by 1 week. Day 1 was a familiarization session; Day 2 was either an experimental session (mindfulness meditation or exercise) or a control session (silent reading); Day 3 and 4 were also either an experimental or control session, depending on participants' unique randomization schedule. Day 2, 3, and 4 were counterbalanced across participants. The executive functioning battery for inhibitory control, working memory and task-switching, along with the psycho-emotional measures were administered before the experimental or control session (pre-test), immediately after the experimental or control session (immediate post-test) and 10 min after the experimental or control session (delayed post-test). Two time points were used to assess the immediate impact of the intervention as well as after a slight delay. This approach was designed to help determine whether any immediate benefits were sustained, or vice-versa, whether any benefits required a delay to emerge. A 10-min delay was selected based on a prior meta-analysis involving acute exercise, which reported that the highest cognitive benefits appear to happen 11–20 min post-exercise (Chang et al., 2012). Further, Luu and Hall (2017) found that the cognitive benefits of mindfulness meditation were not observed at 5 min post-intervention, but were seen at 10 min post-intervention. Figure 1 provides a diagram of the study design.

### Measures

#### ADHD Assessments

Guardians completed two assessments to verify their child's ADHD status: the Vanderbilt Parent Rating Scale (VADPRS; Bard et al., 2013) and the Behavior Rating Inventory of Executive (BRIEF; Shimoni et al., 2012). The results of these assessments are displayed in Supplementary Tables 2, 3. The VADPRS includes all 18 of the DSM-5 criteria for ADHD. The assessment asks guardians to rate the severity of several behaviors on a 4-point Likert scale, ranging from “never” to “very often.” The BRIEF uses guardian ratings on 86-items to provide an understanding of the child's executive functions in routine situations and is another indicator of the presence of ADHD (Shimoni et al., 2012). The questionnaire measures eight different aspects of executive functioning, including inhibition, shifting, emotional control, initiation, working memory, planning and organization, order and organization, and monitoring.

#### Executive Functioning Battery

##### Inhibitory Control

The Stroop Task is a non-invasive measure commonly used to assess inhibitory control in children with ADHD (Yasumura et al., 2014). The task contains a congruent and incongruent portion; we exclusively used the incongruent portion as it aims to specifically target inhibitory control. The Incongruent Stroop Task involves participants reading aloud a list of color words as quickly as they can that are mismatched to the ink color in which they are printed. The participants are instructed to say the color in which the word appears and inhibit their impulse to say the color that is written. For example, if the word *red* is printed in blue ink, the participant should say “blue.” The current study used a paper-based version of the task. Participants had 150 s to read as many words as possible. The outcome measure was the proportion of words read correctly (total words correctly read divided by total words read).

##### Working Memory

The Leiter-3 Reverse Memory Subscale (Roid and Koch, 2017) was used to assess working memory. The task is a complex mental activity that taps into the ability to mentally store and manipulate information. It has been validated in clinical and research settings, and with ADHD populations (Roid and Koch, 2017). The task involves picture matrices of varying sizes (2 × 2; 2 × 6) laid out in front of the participant. The experimenter points to pictures in a pre-established sequence; the participant must then point to the pictures in reverse order of what was presented. The number of pictures that need to be recalled in reverse order gradually increases from 2 to 9. After six errors, the final number of correctly recalled sequences is recorded.

##### Task-Switching

The Trail Making Task (TMT) was used to assess task-switching (Perugini et al., 2000). The TMT contains two parts (A and B). We exclusively used TMT-B, as it measures task-switching (also often referred to as cognitive flexibility). TMT-B has also been validated with ADHD populations (Perugini et al., 2000). The task requires participants to alternate between connecting 25 numbers and letters (i.e., 1, A, 2, B, 3, C, etc.) that are randomly distributed on paper. The outcome variable was the amount of time (in seconds) participants took to complete the task.

#### Psycho-Emotional Assessments

##### Mood

Mood was assessed using Williamson et al.'s (2001) Adapted Version of the Profile of Mood States. Participants indicated the extent to which they were currently experiencing the presented mood on a 5-item Likert scale, ranging from 1 (“not at all”) to 5 (“extremely”). Six positive moods (e.g., happy, energetic, friendly) comprised a “positive mood” construct, and five negative moods (e.g., sad, lonely, unhappy) comprised a “negative mood” construct. Participants' mood states were evaluated at the beginning and end of the protocol on each day of testing.

##### General Self-Efficacy

Chen et al. (2001) General Self-Efficacy scale was used to assess participants' self-efficacy at the beginning and end of the protocol on each day of testing (identical to how mood was assessed). Participants indicated the extent to which they agreed with eight statements relating to general self-efficacy (e.g., *I will be able to successfully overcome many challenges)* on a 5-item Likert scale ranging from 1 (“strongly disagree”) to 5 (“strongly agree”).

#### Physical Activity, Musculoskeletal Fitness, and Body Composition

The Child Physical Activity Questionnaire (PAQ-C; Kowalski et al., 2004) was used to assess children's general physical activity levels during a regular week. Standing long jump distances (index of explosive leg power; Ruiz et al., 2011), and hand grip strength using a handgrip dynamometer (index of musculoskeletal health; Norman et al., 2011) were also collected. Height and weight were collected to calculate body mass index (BMI). Participant scores on these assessments are presented in Supplementary Table 1.

#### Manipulation Checks

##### Heart Rate

During exercise, all participants wore a Fitbit heart rate monitor on their wrist to verify appropriate exertion during biking. Every minute, researchers checked and recorded participants' heart rate in beats per minute (bpm) as indicated on their Fitbit.

##### Ratings of Perceived Exertion

While heart rate was recorded, researchers asked participants to rate their perceived exertion using the Borg CR-10 scale (0 = *not working hard at all*; 5 = *strongly working*; 10 = *extremely strongly*; Williams, 2017). This information was collected to ensure that the participant was not overworking or underworking.

### Experimental Manipulations

#### Exercise Condition

Though there are various forms and intensity levels of exercise, aerobic exercise at moderate levels of intensity appears to consistently benefit cognitive function (Chang et al., 2012; Pontifex et al., 2019). Cycling modalities are currently the most studied, and most effective form of exercise when looking at improvements in cognition (Pontifex et al., 2019). Participants were instructed to cycle continuously for 10 min at a moderate intensity, as determined by 65–85% of the maximum heart rate for their age using the equation 208 – (0.7 × age; Machado and Denadai, 2011). For example, for a 10-year-old, this is between 130 and 170 bpm. Heart rate was checked every minute, and participants were encouraged to peddle faster or slower if they were below or above the designated bpm range, respectively.

#### Mindfulness Meditation Condition

The 10-min mindfulness meditation session was administered using a smartphone application (i.e., Smiling Minds; Smiling Mind, 2015) while participants sat or laid down on a pillow. Smiling Minds is a free mindfulness meditation mobile application based in Cognitive Behavioral Therapy. There is evidence that it can improve—and maintain improvements—in anxiety and depressive symptoms, resilience, and overall mental health in youth (Flett et al., 2019). The specific mindfulness meditation used was called “A Longer Bubble Journey.” It was selected because it was a focused-attention, age-appropriate mindfulness meditation for the current sample. A male voice guided the participant in a body scan while maintaining a focus on their breath. Participants were instructed to attend to specific aspects of their present experience and bodily functioning by following a bubble's journey throughout their body. The mindfulness meditation was delivered through noise-canceling headphones. The experimenters were present in the room during the meditation to ensure adherence to the protocol (e.g., the participant remained listening); however, the lights were dimmed, the experimenters were silent, and participants were left in a corner of the room (the “meditation station”) to approximate privacy.

#### Reading Control Condition

During the reading control condition, participants read age-appropriate magazines for 10 min. A reading control activity has been used in previous exercise intervention research (Pontifex et al., 2013; Drollette et al., 2014) and satisfies conditions for a control group in mindfulness meditation experiments; i.e., the interventions were similar in length and they required the same amount of practice.

### Procedure

Prior to all days of the study, guardians were asked to refrain from giving their child ADHD medication 24 h before coming to the lab. This was done to mitigate potential confounding factors that occur when only some participants are medicated. Prior work has followed a similar procedure (see Medina et al., 2010).

#### Day 1: Familiarization and Questionnaires

Participants and their guardians visited the lab for 45–60 min. Upon arrival, they were briefed about the study protocol and what to expect on each day of their visit. Guardians were provided with a Letter of Information and Consent form; children were provided with a Letter of Information and Assent forms. One researcher helped the guardians complete the two ADHD assessments to verify their child's ADHD status, along with the Demographics Questionnaire and the Medication Use Questionnaire (Supplementary Table 1). At the same time, another researcher helped the child participant complete the PAQ-C (Kowalski et al., 2004) and the physical activity, musculoskeletal fitness and body composition measures. Afterwards, participants were familiarized with the stationary biking procedure that would constitute their exercise session (e.g., how to sit on the bike and pedal, how to put on the wrist-worn Fitbit, how intense to pedal, and how to answer questions related to exercise that would be asked during the study). Participants then sampled the mindfulness meditation audio for 1-min. Lastly, the researchers familiarized the participants with each executive functioning measure. This consisted of showing participants how to correctly complete the Incongruent Stroop Task, TMT, and Leiter-3 Reverse Memory Subscale. The participants then performed each task for ~30 s, or until the tasks were understood.

#### Day 2: Exercise (Experimental)

It should be noted that Day 2 could have involved mindfulness meditation or reading (control) depending on the participant's randomization schedule. If Day 2 involved exercise, participants were required to peddle at a moderate intensity for 10 min on a stationary age-appropriate exercise bike. Given the pre- post-test design of the study, participants first completed the mood and self-efficacy scales (pre-intervention), followed by the executive functioning battery (pre-test). Participants then completed the 10-min biking protocol. Afterwards, participants were re-administered the executive functioning battery (immediate post-test). They were then given a children's magazine to read quietly for 10 min. Finally, they completed the last executive functioning battery (delayed post-test) along with the mood and general self-efficacy assessments (post-intervention). Children were then thanked and returned to their guardians.

#### Day 3: Mindfulness Meditation (Experimental)

Identical to the exercise condition, participants first completed the psycho-emotional assessments and the executive functioning battery. Following, participants completed 10 min of the Smiling Minds guided mindfulness meditation. Afterwards, the executive functioning battery (immediate and delayed post-tests) and psycho-emotional assessments were readministered.

#### Day 4: Reading (Control)

The same procedure as the experimental sessions was followed during the control condition, except participants were asked to read age-appropriate magazines for a 10-min bout instead of engaging in exercise or mindfulness meditation. Once the last measure was collected, guardians and children were debriefed and compensated. Children received $20 per visit (total of $80) and parents received a one-time compensation of $20.

### Statistical Analyses

#### Executive Functioning

All statistical analyses were conducted using SPSS 25. Several repeated measures ANOVAs were conducted with a three-level factor of condition (exercise, mindfulness meditation, reading control), and a three-level factor of time (pre-test, immediate post-test, delayed post-test). Exploratory paired-samples *t*-tests were conducted between pre and immediate post-test executive functioning outcomes within each condition, as well as between pre and delayed post-test executive functioning outcomes within each condition. Bonferroni corrections were applied to account for multiple comparisons.

#### Psycho-Emotional Well-Being

Several repeated measures ANOVAs were conducted with a three-level factor of condition (exercise, mindfulness meditation, reading control), and a two-level factor of time (pre-intervention, post-intervention). For mood, separate analyses were conducted for positive mood and negative mood constructs as outcome variables. For self-efficacy, average general self-efficacy scores were used as the outcome variable. Exploratory paired-samples *t*-tests were conducted between pre-intervention and post-intervention scores within each condition to assess whether there were any changes in psycho-emotional functioning within a condition's session. This practice is aligned with advice that dictates exploratory data analyses should be done to maximize insight into the data set (Komorowski et al., 2016). Bonferroni corrections were applied to account for multiple comparisons.

A one-way ANOVA was conducted on pre-test scores for executive functioning outcomes and psycho-emotional outcomes to confirm no initial condition differences. There were no extreme outliers consistent across variables and conditions using the SPSS step of 1.5 × IQR (interquartile range). Greenhouse Geisser was reported in situations where Mauchly's Test of Sphericity was violated.

## Results

### Manipulation Checks

The average heart rate across participants was 117.5 bpm, which is slightly below 60% of the predicted maximum heart rate for 10–14-year old children. The average rating of perceived exertion was 7.1, indicating a self-reported moderate intensity exercise range.

### Executive Functioning

Descriptive statistics for executive functioning outcomes are provided in Table 1. For inhibitory control, the one-way ANOVA yielded no differences in pre-test scores among conditions *F*_(2, 47)_ = 0.53, *p* = 0.59, η^2^ = 0.02. The repeated measures ANOVA yielded no main effect of time *F*_(2, 30)_ = 0.23, *p* = 0.80, $\eta_{\text{p}}^{2}$ = 0.02, or condition *F*_(2, 30)_ = 2.30, *p* = 0.12, $\eta_{\text{p}}^{2}$ = 0.13; however, there was a significant time by condition interaction *F*_(6, 60)_ = 3.97, *p* = 0.01, $\eta_{\text{p}}^{2}$ = 0.21. Exploratory paired-samples *t*-tests revealed that only the mindfulness meditation condition led to a significant immediate gain in inhibitory control [*t*_(15)_ = −3.04, *p* = 0.01, *d* = 0.86], and this effect was sustained after a delay [*t*_(15)_ = −2.24, *p* = 0.04, *d* = 0.57]. The exercise and control condition did not lead to any changes in inhibitory control (all *t*s < 1.50, all *p*s > 0.16).

**Table 1:** Descriptive statistics for executive functioning tasks.

	**Mindfulness meditation** ***M* (*SD*)**	**Exercise** ***M* (*SD*)**	**Control** ***M* (*SD*)**
**Incongruent stroop (pre-test)**			
**Proportion Correct** Total words read Total errors	0.95 (0.03) 107.56 (29.05) 4.88(3.40)	0.96 (0.02) 115.19 (31.24) 4.38 (3.24)	0.96 (0.04) 109.13 (28.76) 4.37 (3.18)
**Incongruent stroop (immediate post-test)**			
**Proportion Correct** Total words read Total errors	0.98 (0.02) 110.56 (31.50) 2.75 (2.08)	0.96 (0.02) 116.12 (31.72) 4.69 (3.32)	0.95 (0.04) 103.63 (29.46) 5.31 (3.20)
**Incongruent stroop (delayed post-test)**			
**Proportion Correct** Total words read Total errors	0.97 (0.03) 106.63 (29.36) 3.25 (3.10)	0.95 (0.02) 111.88 (26.37) 5.13 (2.42)	0.96 (0.04) 105.69 (29.10) 4.06 (3.34)
**Leiter-3 (pre-test)**			
Number Complete	15.13 (2.47)	15.06 (3.47)	15.69 (1.78)
**Leiter-3 (immediate post-test)**			
Number Complete	16.44 (2.28)	15.69 (2.70)	15.25 (2.27)
**Leiter-3 (delayed post-test)**			
Number Complete	16.89 (1.96)	15.94 (1.95)	16.00 (2.37)
**Trail making task B (pre-test)**			
Time to Completion	103.06 (22.83)	99.28 (45.53)	101.35 (33.66)
**Trail making task B (immediate post-test)**			
Time to Completion	87.84 (31.40)	101.64 (47.82)	97.21 (31.05)
**Trail making task B (delayed post-test)**			
Time to Completion	89.16 (27.89)	92.33 (37.04)	103.87 (38.65)

For working memory, the one-way ANOVA yielded no differences in pre-test scores among conditions *F*_(2, 47)_ = 0.27, *p* = 0.77, η^2^ = 0.01. The repeated measures ANOVA yielded a main effect of time *F*_(2, 30)_ = 7.87, *p* = 0.004, $\eta_{\text{p}}^{2}$ = 0.34, no main effect of condition *F*_(1.45, 21.77)_ = 1.38, *p* = 0.27, $\eta_{\text{p}}^{2}$ = 0.08, and no interaction *F*_(4, 60)_ = 1.52, *p* = 0.21, $\eta_{\text{p}}^{2}$ = 0.09. Exploratory paired-samples *t*-tests revealed that only the mindfulness meditation condition led to a significant immediate gain in working memory [*t*_(15)_ = −3.02, *p* = 0.01, *d* = 0.55], and this effect was sustained after a delay [*t*_(15)_ = −6.22, *p* < 0.001, *d* = 0.78]. The exercise and control condition did not lead to any changes in working memory (all *t*s < −1.42, all *p*s > 0.18).

For task-switching, the one-way ANOVA yielded no differences in pre-test scores among conditions *F*_(2, 47)_ = 0.05, *p* = 0.96, η^2^ = 0.002. The repeated measures ANOVA yielded no main effect of time *F*_(2, 30)_ = 0.87, *p* = 0.43, $\eta_{\text{p}}^{2}$ = 0.06, no main effect of condition *F*_(2, 30)_ = 0.41, *p* = 0.67, $\eta_{\text{p}}^{2}$ = 0.03, and no interaction *F*_(2.08, 60)_ = 1.06, *p* = 0.36, $\eta_{\text{p}}^{2}$ = 0.07. Exploratory paired-samples *t*-tests revealed that only the mindfulness meditation condition led to a significant immediate gain in task-switching [*t*_(15)_ = 2.26, *p* = 0.04, *d* = 0.56], however this effect was not sustained after a delay [*t*_(15)_ = 1.99, *p* = 0.07]. The exercise and control condition did not lead to any changes in task-switching (all *t*s < 0.65, all *p*s > 0.53).

### Psycho-Emotional Well-Being

Descriptive statistics for positive and negative mood outcomes, as well as for general self-efficacy are provided in Table 2. For positive mood, the one-way ANOVA yielded no differences in pre-intervention ratings among conditions *F*_(2, 47)_ = 0.08, *p* = 0.93, η^2^ = 0.003. The repeated measures ANOVA yielded no main effect of time *F*_(1, 15)_ = 2.73, *p* = 0.12, $\eta_{\text{p}}^{2}$ = 0.15, no main effect of condition *F*_(1, 15)_ = 1.10, *p* = 0.35, $\eta_{\text{p}}^{2}$ = 0.07, and no interaction *F*_(2, 30)_ = 1.56, *p* = 0.23, $\eta_{\text{p}}^{2}$ = 0.09. Exploratory paired-samples *t*-tests revealed the exercise condition led to a significant positive change in mood from pre- to post-intervention *t*_(15)_ = −2.700, *p* = 0.02, *d* = 0.35, whereas the mindfulness meditation and reading control conditions did not (*t*s < −1.03, *p*s > 0.32). For negative mood, the one-way ANOVA yielded no differences in pre-intervention ratings among conditions *F*_(2, 47)_ = 0.19, *p* = 0.83, η^2^ = 0.01. The repeated measures ANOVA yielded no main effect of time *F*_(1, 15)_ = 0.43, *p* = 0.52, $\eta_{\text{p}}^{2}$ = 0.03, no main effect of condition *F*_(2, 30)_ = 0.69, *p* = 0.51, $\eta_{\text{p}}^{2}$ = 0.04, and no interaction *F*_(2, 30)_ = 0.06, *p* = 0.94, $\eta_{\text{p}}^{2}$ = 0.004. Exploratory paired-samples *t*-tests revealed no differences within each condition between pre- and post-intervention negative mood ratings (all *t*s < 0.63, all *p*s > 0.54).

**Table 2:** Descriptive statistics for mood and general self-efficacy outcomes.

	**Mindfulness meditation** ***M* (*SD*)**	**Exercise** ***M* (*SD*)**	**Control** ***M* (*SD*)**
**Ratings of Positive Mood—**Pre-intervention	3.51 (1.06)	3.61 (.82)	3.63 (0.99)
**Ratings of Positive Mood—**Post-intervention	3.54 (1.14)	3.91 (.87)	3.74 (0.84)
**Ratings of Negative Mood—**Pre-intervention	1.65 (0.51)	1.63 (.44)	1.55 (0.48)
**Ratings of Negative Mood—**Post-intervention	1.59 (0.45)	1.59 (.55)	1.54 (0.45)
**Ratings of General Self-Efficacy—**Pre-intervention	3.72 (0.76)	3.65 (.77)	3.82 (0.81)
**Ratings of General Self-Efficacy—**Post-intervention	3.87 (0.73)	3.82 (.74)	3.91 (0.87)

For general self-efficacy, the one-way ANOVA yielded no differences in pre-intervention ratings among conditions *F*_(2, 47)_ = 0.18, *p* = 0.84, η^2^ = 0.01. The repeated measures ANOVA yielded no main effect of time *F*_(1, 15)_ = 4.09, *p* = 0.06, $\eta_{\text{p}}^{2}$ = 0.21, no main effect of condition *F*_(2, 30)_ = 1.04, *p* = 0.37, $\eta_{\text{p}}^{2}$ = 0.07, and no interaction *F*_(2, 30)_ = 0.28, *p* = 0.76, $\eta_{\text{p}}^{2}$ = 0.02. Exploratory paired-samples *t*-tests revealed similar results to positive mood in that the exercise condition led to a significant positive change in general self-efficacy from pre- to post-intervention *t*_(15)_ = −2.29, *p* = 0.04, *d* = 0.22, whereas the mindfulness meditation and reading control conditions led to no changes (all *t*s < −1.45, all *p*s > 0.17).

## Discussion

This study investigated whether acute exercise and mindfulness meditation impact executive functioning and psycho-emotional well-being in children with ADHD. The efficacy of these two behavioral interventions was also compared. For executive functioning, only acute mindfulness meditation increased performance on tasks of inhibitory control, working memory and task-switching. The benefits of mindfulness meditation were evident both immediately after the intervention as well as after a 10-min delay for inhibitory control and working memory. Task-switching performance only showed improvements immediately after mindfulness meditation but not after a delay. In terms of psycho-emotional well-being, exploratory *t*-tests revealed that only acute exercise increased positive mood and general self-efficacy ratings from pre- to post-intervention. Negative mood was not impacted by either acute exercise or mindfulness meditation. Overall, these findings suggest that an acute bout of mindfulness meditation may better support executive functioning than acute exercise among children and youth with ADHD. Considering prior work has demonstrated that acute exercise generally leads to improvements in executive functioning (Pontifex et al., 2019), further research is needed to substantiate these findings in children with ADHD. With respect to psycho-emotional well-being, acute exercise might be a superior strategy to support positive changes in mood and general self-efficacy. Our study is strengthened by using a within-subject pre-test post-test experimental design, which is considered the gold-standard for testing the acute effects of exercise on executive functioning (Pontifex et al., 2019). However, with this design, it is possible that one intervention influenced the effect of the other. Future research should test this possibility using a between-subject design.

To our knowledge, this is the first experimental study that provides evidence for improved executive functioning in children and youth with ADHD following just one mindfulness mediation session. Finding that inhibitory control is improved following one mindfulness meditation session is particularly notable, given that youth with ADHD struggle in this domain (Antshel et al., 2014). These results are aligned with previous work showing that mindfulness meditation interventions can support inhibitory control in typically developing adults and undergraduates (Wenk-Sormaz, 2005; Zylowska et al., 2008; Luu and Hall, 2017). There are several potential explanations for these findings. First, mindfulness meditation has been shown to support response de-automatization, which is the ability to respond to situations in a flexible way consistent with what a situation demands instead of relying on habitual automatic responses (Wenk-Sormaz, 2005; Kang et al., 2013). This is especially helpful for individuals with ADHD, as they often struggle to inhibit prepotent responses that may not be conducive to the current situation. Second, mindfulness meditation may support reductions in physiological stress by inducing relaxation (Hölzel et al., 2011), which in turn may help counteract inappropriate impulses. Indeed, mindfulness meditation has been shown to induce physiological relaxation through reductions in heart and respiratory rate (Hölzel et al., 2011). This could potentially have a direct impact on individuals with ADHD considering the disorder is often linked to higher average heart rate, especially for those receiving stimulant medication (Mick et al., 2013). And third, mindfulness meditation is associated with decreased amygdala activation—the “emotional” processing center of the brain, which is theorized to be the result of the prefrontal cortex (PFC) exerting top-down control over the structure (Hölzel et al., 2011; Tang et al., 2015). The PFC is involved in higher order executive functions, such as inhibitory control, working memory, emotion regulation, and communication (Arnsten and Li, 2005). Simply put, mindfulness meditation may enable the “logical” PFC to overtake the influence of the “emotional” amygdala, thereby enabling meditators to better inhibit expression of automatic emotional responses (Hölzel et al., 2011). Because ADHD is partly characterized by dysfunction of the PFC as well as emotional dysregulation, and deficits in PFC functioning are related to decreased inhibitory control (Arnsten and Li, 2005), mindfulness meditation increasing PFC function may help compensate for these deficits, thus improving inhibitory control (Tang et al., 2015). Whether inhibitory control improvements occur because mindfulness meditation facilitates response de-automatization, relaxation, improved PFC functioning, or a combination of these processes, is unclear; further research is needed to clarify these mediating processes.

This study also provides initial evidence that acute mindfulness meditation may help support the working memory of children with ADHD. This finding is consistent with studies showing working memory improvements following extensive mindfulness meditation training (i.e., weeks or months) with healthy populations (Zeidan et al., 2010), as well as with ADHD populations (Bachmann et al., 2016; Janssen et al., 2019). These results may be explained by the Default Mode Network (DMN). The DMN is a brain network that includes midline neurological structures like the medial PFC, posterior cingulate cortex, and inferior parietal lobule. The network is usually active during rest, and inactive during task performance. ADHD is sometimes conceptualized as a result of DMN disruption (Bachmann et al., 2016). Those with the disorder display atypical connectivity within the DMN, as well as between the DMN and other cortical structures. Additionally, the DMN can remain active in individuals with ADHD when it should be inactive, resulting in constant disruption of other neural networks. Activation in parts of the DMN has been shown to correspond with improved working memory following mindfulness meditation (Bachmann et al., 2016). This may occur because meditators can exert greater control over DMN activity than non-meditators (Brewer et al., 2011; Tang et al., 2015), and the DMN appears to relax following mindfulness meditation. The current study's finding that working memory task performance improved following mindfulness meditation may be rooted in greater DMN regulation, thereby resulting in enhanced functioning of brain circuits related to working memory. Future neuroimaging studies using the current experimental design could help elucidate the interplay between acute mindfulness meditation, the DMN, and the corresponding impact on working memory function.

Task-switching performance also improved following acute mindfulness meditation, although this result was only observed immediately after the intervention and disappeared after a delay. This finding is in line with previous work showing mindfulness interventions enhance task-switching and attentional processes (Luu and Hall, 2017; Lo et al., 2020). However, in these prior studies, mindfulness meditation interventions took place over longer periods of time (6 and 8 weeks, respectively). The current study provides novel evidence for the benefits of a single 10-min bout of mindfulness meditation on immediate task-switching performance. Despite the immediate benefits of mindfulness meditation on task-switching, these findings disappeared after a delay. Some researchers suggest sustained mindfulness meditation practice is required to have a long-term impact on cognitive flexibility (e.g., Johnson et al., 2015). Regardless, the immediate benefit of acute mindfulness meditation on task-switching performance is an important finding given the challenges that many children and youth with ADHD face with cognitive flexibility.

Contrary to hypotheses, acute mindfulness meditation did not impact mood or general self-efficacy. This contrasts with several studies that found psycho-emotional measures were improved by brief mindfulness meditation (e.g., Johnson et al., 2015; Luu and Hall, 2017; Edwards et al., 2018). However, these findings were obtained from research with neurotypical adults, and thus may not reflect functioning of children or individuals with ADHD. Another reason for the discrepancy may have been because many young people, especially those with ADHD, report that they struggle with mindfulness meditation (Murrell et al., 2015; Keller et al., 2017). Mindfulness meditation practices could, by their very nature, increase psychological distress and ADHD symptoms, as they bring attention to one's inattention (Murrell et al., 2015). Although this was not formally assessed in the current study, experimenters reported observing that many participants seemed resistant to the prospect of mindfulness meditation. This apparent negativity could explain why the increase in psycho-emotional well-being observed in previous research was not present in the current work. Future research should measure participants' tolerance or enjoyment of various treatments to determine whether these factors play a role in the psycho-emotional efficacy of interventions.

Surprisingly, there were no improvements in any executive functioning domain following acute exercise. This is contrary to prior work which has shown that acute exercise can enhance performance in inhibitory control, working memory, and tasking switching among children with ADHD (Pontifex et al., 2013; Drollette et al., 2014; Benzing et al., 2018). One explanation for this discrepancy is the duration of exercise used across studies. The current study's exercise manipulation was 10 min long, whereas the aforementioned studies that have yielded positive effects following acute exercise used intervention durations ranging from 15 to 40 min.

Another explanation for the discrepancy in findings relative to prior research may reflect the type of exercise used across studies. Prior studies showing benefits of acute exercise on executive functioning have often used cognitively engaging exercise, which includes the addition of a task that requires goal-directed behavior (Crova et al., 2013; Benzing et al., 2018). Examples of this include individual and team sports, which require the integration of exercise and rapid cognitive processing to successfully plan and anticipate behavior, employ strategies, and adapt to changing task demands. The cognitive demand of complex exercise likely increases cognitive engagement, which refers to the allocation of cognitive resources necessary to master and complete a difficult task (Schmidt et al., 2015). This, in turn, may support the enhanced functioning of those cognitive processes on subsequent cognitive tasks due to the pre-activation of the same brain regions. In support of this, Schmidt et al. (2015) demonstrated that participants engaging in cognitively demanding exercise showed gains in task-switching, while participants engaging in simple aerobic exercise did not. Furthermore, studies using other forms of cognitively engaging exercise, such as exergaming (Benzing et al., 2016) and the use of playful exercise games that target executive functions (Jäger et al., 2014), demonstrated improvements in inhibitory control, cognitive flexibility and set-shifting. Although cognitively engaging exercise may require a similar form of thinking and skill, which may then have lingering transfer effects on following executive function tasks (Best, 2010), repetitive aerobic exercises (e.g., stationary biking, treadmill walking, or running) typically do not require thoughtful consideration during movement to achieve a goal. Thus, aerobic exercise may be less cognitively demanding and confer fewer benefits to executive functioning. In the current study, 10 min was selected as the stimulus duration and cycling as the exercise modality based on combined recommendations by Chang et al. (2012) and Pontifex et al. (2019) that suggested these were the most likely paradigms to promote executive functioning. In retrospect, these recommendations were not necessarily made specifically for children with ADHD, who may require a different duration and modality of exercise to cognitively benefit from acute exercise.

With respect to psycho-emotional well-being however, acute exercise did appear to yield benefits for children and youth with ADHD. In particular, ratings of both positive mood and general self-efficacy improved following 10 min of exercise. This supports previous work showing that engaging in exercise significantly improves ratings of mood and self-efficacy in both neurotypical children and adults, as well as in those with ADHD (e.g., Cornelius et al., 2017). However, this study is among the few that has demonstrated psycho-emotional improvements with acute exercise for children with ADHD. Identifying whether acute exercise can promote positive mood and self-efficacy for children with ADHD can help support the call for more integration of acute exercise in public domains, such as schools and classrooms. This is especially helpful for children with ADHD who are at heightened risk for low self-efficacy and mood-related disorders (Gordon-Lipkin et al., 2018), which can negatively impact many facets of everyday life including academic learning.

To the authors' knowledge, this was the first study to evaluate the relative contributions of acute exercise and mindfulness meditation in supporting executive functioning and psycho-emotional well-being among children with ADHD. As mentioned, prior studies have either been systematic reviews with no direct empirical comparisons between exercise and mindfulness meditation (Herbert and Esparham, 2017), or have been done exclusively in neurotypical younger adults (Van Der Zwan et al., 2015; de Bruin et al., 2016; Luu and Hall, 2017) or neurotypical older adults (Håkansson et al., 2017). There has been no work to date comparing these interventions in the context of children with ADHD. The current findings provide preliminary evidence for a potentially greater benefit of acute mindfulness meditation over acute exercise for executive functioning among children and youth with ADHD. In contrast, the study's findings suggest that when it comes to psycho-emotional well-being, acute exercise may be more conducive to supporting positive mood and general self-efficacy among children and youth with ADHD.

The dichotomy in the efficacy of these interventions for executive functioning may relate to the mechanisms underlying their effect. Although the current study cannot pinpoint the exact mechanisms underlying the observed benefit of mindfulness meditation on executive functioning, the results suggest that the duration and modality of mindfulness meditation that was employed either supported response de-automatization, elicited relaxation, improved PFC functioning or a combination of these processes. In contrast, the acute exercise stimulus that was employed was insufficient to yield executive functioning benefits potentially due to duration or a lack of cognitive complexity or to other factors that were not considered.

The dichotomy in the efficacy of these interventions for psycho-emotional well-being may be rooted in a combination of discrepancies in psychological and physiological responsiveness. Although there is some evidence that boys with ADHD hold negative views about exercise (Harvey et al., 2009), their negativity is often rooted in social comparison and less proficient movement skills relative to their peers without ADHD. Given that the study's participants engaged in isolated exercise without peers, negative social associations may have been mitigated. However, mindfulness meditation has been noted as potentially drawing attention to children's ADHD symptoms of inattention and may thus be more likely to undermine any acute psycho-emotional benefits (Murrell et al., 2015). Furthermore, exercise, even in acute bouts, has been shown to alter aspects of neurochemistry (Pontifex et al., 2013; Cerrillo-Urbina et al., 2015). Long-term mindfulness meditation training has also been shown to have neuroanatomical and neurochemical effects (Tang et al., 2015), but it is unclear what the acute benefits of mindfulness meditation are on neurophysiology and corresponding psycho-emotional well-being. The benefits of acute exercise for psycho-emotional functioning are currently more robust from both a psychological and physiological perspective compared to acute mindfulness meditation and may thus help explain why acute exercise yielded psycho-emotional benefits in the current sample whereas acute mindfulness meditation did not.

### Implications

One of the most important implications of the current work is that an acute mindfulness meditation session can promote inhibitory control, which is an area where many children with ADHD struggle (Antshel et al., 2014). Many individuals with ADHD struggle to sit for long periods of time (Murrell et al., 2015), which makes long-term mindfulness meditation interventions difficult for them (as extended sitting is an integral part of the practice). Thus, observing that merely 10 min of mindfulness meditation supports inhibitory control may increase the feasibility and reach of such interventions, as individuals with ADHD may still reap inhibitory control benefits without sitting for long periods.

This study also adds to the literature assessing how mindfulness-based interventions affect children and youth with ADHD. Specifically, our findings converge with the research that has determined acute mindfulness-based interventions can enhance aspects of functioning (e.g., Luu and Hall, 2017; Carsley and Heath, 2018; Edwards et al., 2018). It uniquely contributes to the literature, however, by experimentally demonstrating that key aspects of executive functioning (i.e., inhibitory control and working memory) can be supported in children and youth with ADHD after engaging in just one mindfulness meditation session.

Regarding psycho-emotional well-being, the current study provides preliminary evidence for the benefit of acute exercise for promoting positive mood and general self-efficacy for children and youth with ADHD. Due to the length of the exercise bout, these results may increase the feasibility of an acute exercise intervention for children and youth with ADHD, providing guardians and teachers the option to incorporate just 10 min of exercise to assist children emotionally, while simultaneously reducing their sedentary behavior.

In summary, the current work suggests that acute exercise and mindfulness meditation can have differential positive effects on executive functioning and psycho-emotional well-being in children and youth with ADHD. These results provide a nuanced perspective into how behavioral interventions could be personalized according to a child's needs. Specifically, depending on the particular needs of a certain child or group of children and youth with ADHD, teachers, clinicians, or parents could combine, integrate, and customize behavioral activities (e.g., acute exercise vs. acute mindfulness meditation) according to children's dynamic executive functioning or psycho-emotional needs.

### Limitations

This study did not have an age-matched, neurotypical control group, which limits the ability to compare and contrast the implications of acute exercise and mindfulness meditation on executive functioning and psycho-emotional well-being in typical vs. atypical development. Also, only children with a formal ADHD diagnosis were recruited. There is a high degree of variability in terms of how ADHD is diagnosed. It is possible that the study's sample had meaningful differences in terms of ADHD diagnoses, which could ultimately affect the study's claims. Ideally, an independent diagnostic interview would be conducted to verify ADHD status, however that was beyond the scope of study resources.

Like all populations, children with ADHD vary in trait mindfulness (i.e., baseline levels of mindfulness; Smalley et al., 2009). The present study did not assess trait mindfulness, as we were interested in the effect of state-mindfulness changes (i.e., temporary changes in mindfulness) elicited by the intervention. Nonetheless, future research should include a measure of trait mindfulness, as trait mindfulness levels often differ from state mindfulness (Bravo et al., 2018). Measuring trait mindfulness would allow researchers to determine how previous levels of mindfulness impact the efficacy of the intervention. Similarly, future research should include a mindfulness manipulation check. The Toronto Mindfulness Scale (Lau et al., 2006), for example, is a measure commonly used to check participant engagement during mindfulness sessions in research (e.g., Miyahara et al., 2020). Data obtained by this measure would provide insight into how variance in mindfulness engagement during the intervention related to experimental outcomes.

It is important to mention that some of the participants were on medication for ADHD. Although participants were asked to refrain from using their medication for 24-h prior to testing days, it is possible that those on medication differed in their performance compared to those who were not medicated. The sample size was not large enough to conduct individual difference analyses based on medication use. However, at least with respect to the effects of exercise, prior research (Medina et al., 2010) has shown that medication use does not have an impact on the responsiveness of children with ADHD to exercise interventions. Similar support from prior research for mindfulness meditation interventions does not currently exist; it would thus be helpful for future work to investigate the differential impact of medication use on mindfulness meditation responsiveness in children and youth with ADHD.

### Conclusion

In closing, this study adds to an important body of existing research that supports the efficacy of long-term mindfulness meditation training by demonstrating that mindfulness meditation does not need to be a daily (or even weekly) practice to be effective; rather, an acute session could help executive functioning deficits among children and youth with ADHD. Furthermore, the results contribute novel evidence for the psycho-emotional benefits of acute exercise for children with ADHD. These findings provide valuable insight into how parents, teachers, and clinicians can begin personalizing behavioral strategies depending on the fluctuating cognitive and psycho-emotional needs of children with ADHD to best support different domains of functioning.

## Data Availability Statement

The raw data supporting the conclusions of this article will be made available by the authors, without undue reservation.

## Ethics Statement

This study involving human participants was reviewed and approved by Western University Research Ethics Board. Written informed consent to participate in this study was provided by the participants' legal guardian/next of kin. Verbal assent was obtained from the participants' themselves.

## Author Contributions

HB and MG contributed to all the aspects of the manuscript process including study conception, study design, data collection, and analysis as well as manuscript preparation. MO and JG contributed to the study design, data analysis, and manuscript preparation. BF contributed to the study conception and design, data analysis, and manuscript preparation. All authors contributed to the article and approved the submitted version.

## Conflict of Interest

The authors declare that the research was conducted in the absence of any commercial or financial relationships that could be construed as a potential conflict of interest.
