# Supplementary material for: The Differential Impact of Acute Exercise and Mindfulness Meditation on Executive Functioning and Psycho-Emotional Well-Being in Children and Youth With ADHD
Source: Front Psychol. 2021 Jun 14;12:660845. doi: 10.3389/fpsyg.2021.660845 (PMC8236645; doi:10.3389/fpsyg.2021.660845)
Supplement: Supplementary file 1 [file Table_1.DOCX]

Supplementary Material

# Supplementary Tables

## Supplementary Table 1

*Participant Demographics and Medication Use*

| **Characteristic** | **Value** |
| --- | --- |
| Age of Child (years), mean (SD) | 11.38 (1.50) |
| Age of Guardian (years), mean (SD) | 47.56 (7.98) |
| Sex of Participant |  |
| Male | 11 |
| Female | 5 |
| Sex of Guardian |  |
| Male | 7 |
| Female | 9 |
| Height (cm), mean (SD) | 144.01 (13.79) |
| Weight (kg), mean (SD) | 47.15 (16.16) |
| Grip Strength (right; lbs) mean (SD) | 35.91 (11.08) |
| Grip Strength (left; lbs) mean (SD) | 33.73 (9.77) |
| Standing Long Jump (cm), mean (SD) | 125.72 (29.30) |
| PAQ-C Scores | 1.86 (1.00) |
| Guardian’s Education Level (n) |  |
| Some high school, no diploma | 1 |
| High school graduate, diploma or the equivalent | 0 |
| Some college credit, no degree | 2 |
| Trade/technical/vocational training | 2 |
| Associate degree | 1 |
| Bachelor’s degree | 5 |
| Master’s degree | 4 |
| Professional degree | 1 |
| Guardian’s Employment (n) |  |
| Employed for wages | 15 |
| Self-employed | 0 |
| Out of work | 0 |
| Homemaker | 1 |
| Household Income |  |
| Prefer not to say | 2 |
| < $30 000 | 0 |
| $30 000 - $40 000 | 1 |
| $40 000 - $50 000 | 2 |
| $50 000 - $60 000 | 1 |
| $60 000 - $70 000 | 2 |
| $70 000 - $80 000 | 3 |
| $80 000 - $90 000 | 2 |
| $90 000 - $100 000 | 0 |
| > $100 000 | 3 |
| Age of ADHD diagnosis (n) |  |
| Unsure | 1 |
| 4 | 1 |
| 5 | 0 |
| 6 | 2 |
| 7 | 4 |
| 8 | 3 |
| 9 | 4 |
| 10 | 0 |
| 11 | 1 |
| ADHD Subtype (n) |  |
| Predominantly inattentive | 3 |
| Predominantly hyperactive | 1 |
| Combined subtype | 3 |
| Unsure/no diagnosis given | 9 |
| Currently Taking Medication |  |
| No response | 1 |
| Yes | 9 |
| No | 6 |
| Other Diagnosis Present |  |
| Yes | 6 |
| No | 10 |
| Medicated for Another Diagnosis |  |
| Yes | 2 |
| No | 14 |

## Supplementary Table 2

*Vanderbilt Parent Rating Scale*

|  | *N* | % of Sample |
| --- | --- | --- |
| Inattentive Only |  |  |
| Clinically significant | 5 | 31.25 |
| Not clinically significant | 11 | 68.75 |
| Hyperactive/Impulsive Only |  |  |
| Clinically significant | 1 | 6.25 |
| Not clinically significant | 15 | 93.75 |
| Combined Subtype |  |  |
| Clinically significant | 7 | 43.75 |
| Not clinically significant | 9 | 56.25 |
| Oppositional-defiant disorder |  |  |
| Clinically significant | 8 | 50.00 |
| Not clinically significant | 8 | 50.00 |
| Conduct disorder |  |  |
| Clinically significant | 1 | 6.25 |
| Not clinically significant | 15 | 93.75 |
| Anxiety |  |  |
| Clinically significant | 1 | 6.25 |
| Not clinically significant | 15 | 93.75 |
| Performance |  |  |
| Clinically significant | 13 | 81.25 |
| Not clinically significant | 3 | 18.75 |

## Supplementary Table 3

*Behavior Rating Inventory of Executive Function (BRIEF)*

|  | N | % of Sample |
| --- | --- | --- |
| Inhibition |  |  |
| Clinically significant | 7 | 43.75 |
| Not clinically significant | 9 | 56.25 |
| Self-Monitor |  |  |
| Clinically significant | 10 | 62.50 |
| Not clinically significant | 6 | 37.50 |
| Behaviour regulation index |  |  |
| Clinically significant | 10 | 62.50 |
| Not clinically significant | 6 | 37.50 |
| Shift |  |  |
| Clinically significant | 13 | 81.25 |
| Not clinically significant | 3 | 18.75 |
| Emotional Control |  |  |
| Clinically significant | 9 | 56.25 |
| Not clinically significant | 7 | 43.75 |
| Emotion regulation index |  |  |
| Clinically significant | 10 | 62.50 |
| Not clinically significant | 6 | 37.50 |
| Initiate |  |  |
| Clinically significant | 8 | 50.00 |
| Not clinically significant | 8 | 50.00 |
| Working Memory |  |  |
| Clinically significant | 10 | 62.50 |
| Not clinically significant | 6 | 37.50 |
| Planning |  |  |
| Clinically significant | 8 | 50.00 |
| Not clinically significant | 8 | 50.00 |
| Task Monitoring |  |  |
| Clinically significant | 11 | 68.75 |
| Not clinically significant | 5 | 31.25 |
| Organization |  |  |
| Clinically significant | 8 | 50.00 |
| Not clinically significant | 8 | 50.00 |
| Cognitive regulation index |  |  |
| Clinically significant | 13 | 81.25 |
| Not clinically significant | 3 | 18.75 |
